# The Efficacy of a Calamansi-Containing Energy Drink on Running Performance and Recovery in NCAA Division I Middle-Distance Runners: A Preliminary Study

**DOI:** 10.3390/ijerph182111023

**Published:** 2021-10-20

**Authors:** Abdullah B. Alansare, Josh Hayman, Jung-Min Lee, Myong-Won Seo, Deoksu Yoo, Hyun Chul Jung

**Affiliations:** 1 Department of Exercise Physiology, College of Sport Sciences and Physical Activity, King Saud University, King Khalid Rd., Riyadh 11543, Saudi Arabia; aalansare@ksu.edu.sa; 2 Athletic Department, Mercer University, 1501 Mercer University Dr., Macon, GA 31207, USA; hayman_jt@mercer.edu; 3 Department of Physical Education, College of Physical Education, Kyung Hee University, Global Campus, 1732 Deokyoungdaero, Giheung-gu, Yongin-si 17014, Korea; jungminlee@khu.ac.kr; 4 Department of Exercise Science, David B. Falk College of Sport and Human Dynamics, Syracuse University, Syracuse, NY 13244, USA; mseo04@syr.edu; 5 Department of Taekwondo, College of Physical Education, Kyung Hee University, 1732 Deokyoungdaero, Giheung-gu, Yongin-si 17014, Korea; powerds@khu.ac.kr; 6 Department of Coaching, College of Physical Education, Kyung Hee University, Global Campus, 1732 Deokyoungdaero, Giheung-gu, Yongin-si 17014, Korea

**Keywords:** running, performance, recovery, energy drink, caffeine

## Abstract

This study examined the effects of a non-caffeinated energy drink (ED) that contained calamansi juice, glucose, and taurine on 3-km running performance and recovery. Eleven NCAA Division I middle-distance runners (20.8 ± 1.5 years old) were randomly assigned to consume either the ED or a placebo drink 60 min before 3-km running on a 400-m official track. Performance time and speed were recorded every 500-m interval. Recovery blood lactate concentration (BLC), systolic (SBP), diastolic blood pressure (DBP), and heart rate (HR) were measured at baseline, 60-min after ingesting the drinks, and post-running measurements were performed at 1-min, 5-min, and 10-min. Repeated analysis of variance and paired *t*-test were applied to examine the effects of time, trials, and their interaction on performance and recovery. Statistical significance was set a priori at *p <* 0.05. No significant difference was observed in performance time and speed between trials (*p <* 0.05). No interaction effect was found on performance time, speed, recovery BLC, DBP, and HR (*p <* 0.05). However, an interaction effect for trial by time was observed on SBP (*p* = 0.01). Recovery SBP continues to decrease from 5-min to 10-min in the ED trial (∆ = −13.9 mmHg) and slightly increased in the placebo trial (∆ = 1.1 mmHg). This study suggests that acute consumption of a calamansi-containing ED can positively impact the SBP recovery but not running performance. Further studies are needed to examine the acute and chronic effects of this ED on exercise performance and recovery among different populations.

## 1. Introduction

Energy drinks (EDs) are often consumed as nutritional ergogenic aids to improve exercise performance and recovery [1]. These benefits are attributed to the sole and/or combined effects of EDs’ ingredients. Particularly, caffeine alone or when combined with other ingredients such as taurine, carbohydrate, and vitamins (e.g., B group, C) has been suggested to be the primary component that enhances exercise performance [2,3]. Yet, recent evidence indicates that caffeinated EDs not only can delay recovery [4,5] but can also adversely impact various health outcomes such as toxicity, insomnia, cardiac arrhythmia, and fibrillation [6,7]. Due to these adverse impacts, advocates to ban EDs sale, especially caffeinated ones, has already been established [8].

An alternative strategy is to replace caffeine with other substances that may provide the intended ergogenic influence on exercise performance and recovery without similar adverse impacts. Specifically, Taurine is an amino acid that exerts favorable effects on insulin activity, glycemic regulation, lactate concentration, and anti-inflammation [9,10,11]. Administration of Taurine was found to have a favorable influence on exercise-induced fatigue and exercise performance and recovery [12,13]. Similarly, glucose is the basic form of carbohydrate that can enhance the production of the molecular unit of currency (adenosine triphosphate, ATP), reduced exercise-induced fatigue, and improve exercise performance [14,15]. In fact, both Taurine and glucose have commonly been added to EDs [1] due to their beneficial effects on exercise performance and recovery. Thus, Taurine and glucose may be optimal surrogates to caffeine in EDs.

A crucial determinant that may limit exercise performance and recovery and accelerate exercise-induced fatigue even if EDs were consumed is reactive oxygen species (ROX) [16,17]. The detrimental impacts of ROX are usually observed following high intensity or prolonged exercise [16]. These harmful effects include decreasing muscle force production and damaging lipids and proteins in the contracting myocytes, all of which can impact exercise performance and recovery [18]. Antioxidants such as dietary nitrate supplementations and vitamins have been proposed to mitigate the unfavorable effects of ROX during and following exercise [17]. Of particular interest, vitamin C is one suggested antioxidant to blunt biological, oxidative changes [17], which could potentially delay exercise-induced fatigue and enhance exercise recovery. Moreover, vitamin C can enhance carnitine biosynthesis and bioavailability [19], which is known for its essential role in transporting fatty acid into mitochondria for ATP production by B-oxidation [20]. Thus, vitamin C consumption may also contribute to improving exercise performance via promoting fatty acid oxidation. Uniquely, Calamansi is a citrus fruit that grows abundantly in East Asia and is rich with antioxidant agents particularly vitamin C. Altogether, the combination of Taurine, glucose, and vitamin C as a form of ED may result in a beneficial influence on exercise performance and recovery. Vitamin C can be extracted from natural sources such as citrus fruits.

To the best of our knowledge, only one study examined the ergogenic effect of an ED that contained Taurine, glucose, and calamansi juice on exercise performance and recovery in female basketball players (*n* = 6; 21.5 ± 1.9 years old) [21]. In a randomized controlled trial design, the authors have evaluated the influence of the ED versus placebo drink on muscular power, agility, and anaerobic using vertical jump, 10 m × 5 shuttle run, Wingate tests, respectively. Their findings suggested that the ED resulted in higher muscular power and faster fuel replenishment compared to the placebo trial. These improvements may be explained by quick absorption of the ingested Taurine and glucose which can enhance insulin activity, maintain blood glucose concentration, and spare endogenous glycogen stores, respectively [11,22]. Yet, the efficacy of such EDs on endurance performance and recovery remains to be explored.

Therefore, the purpose of this study was to examine the acute effects of ED that contained calamansi juice, taurine, and glucose on 3-km running performance and recovery in NCAA division I middle-distance runners. It was hypothesized that the ED would improve 3km running performance and physiological and metabolic recovery including heart rate (HR), blood pressure (BP), and blood lactate concentration (BLC) compared to a placebo drink.

## 2. Materials and Methods

### 2.1. Study Design

**Figure 1:**
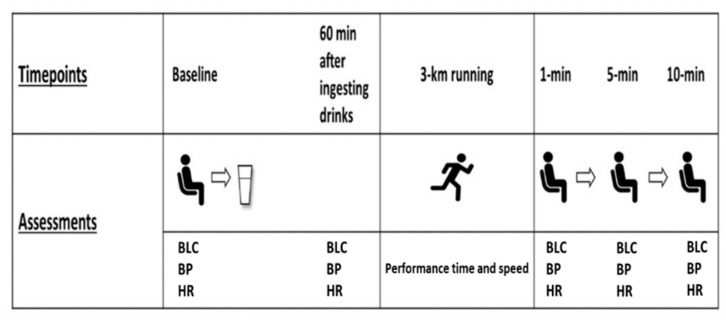
Study procedure BLC; blood lactate concentration, BP; blood pressure, HR; heart rate.

This study was conducted in a randomized, double-blinded, and crossover design. All participants visited the laboratory on two different occasions at the same time of the day, separated by a 7-day washout period to ensure the carry-out effects were eliminated. All visits took place between mornings and afternoons. During visit one, participants underwent baseline measurements including body height and mass, body mass index, body fat percentage, resting HR, BP, and BLC. Then, participants were randomly assigned to the ED or placebo drink trial. The assigned drink was provided to participants who were instructed to consume it 60 min prior to their running start. Afterward, participants performed 3-km running on a 400-m official, outdoor track. Performance time and speed were recorded for the ED and placebo drink trials. Recovery HR, BP, and BLC were measured at baseline, 60-min after the ingestion of the ED/placebo drink, 1-min, 5-min, and 10-min post-running (Figure 1). After a 7-day washout period, participants performed the same experimental procedure as their first trial with consuming the ED or placebo drink upon their supplementation at the first trial. During the washout period, participants were instructed to carry on their normal activities and training.

### 2.2. Participants

Eleven collegiate distance runners who were involved in the National Collegiate Athletic Association (NCAA) Division I voluntarily participated in this study. All participants were engaged in their regular training (twice/day for five days a week). The inclusion criteria of this study were: (a) collegiate NCAA Division I male and female distance runners, (b) had no musculoskeletal injuries in the last three months, (c) were not taking any vitamin supplementation such as vitamin A, B, C, D, E for the last three months, and (d) regularly participated in track and field training. In accordance with the seventh version of the Declaration of Helsinki ethical principles (2013), all eligible participants were provided with the study information including the purpose of the study, study procedure, possible benefits, and risks. All participants provided informed consent. This study was approved by the institutional review board (IRB) at the University of Louisiana at Monroe.

### 2.3. Body Composition

Standing body height and weight were measured with a standardized balance beam scale (DetectoTM, Model 439, Webb City, MO, USA) to nearest 0.1 cm and 0.1 kg, respectively. The measurements were performed while participants wore light cloth with bare feet. Body mass index was computed by dividing weight (kg) by squared height (m^2^). Body fat percentage was estimated from skinfold thickness using Lange skinfold caliper (FAB12-1110, Beta technology, Santa Cruz, CA, USA). The three-site skinfold method was applied. Chest, abdominal, and thigh skinfold thickness were measured for males, and triceps, suprailliac, and thigh for females. The measurements were completed twice from the right side of each participant. The criterion for the agreement between the two measures was within 5%. Thereafter, body density was calculated by Jackson and Pollock’s equation, and body fat percentage was computed by Siri’s equation, respectively [23,24].

### 2.4. Blood Lactate Concentration Measurement

Seated BLC was evaluated five times as follows: at baseline, 60-min after the ingestion of the ED/placebo drink, post-running at 1-min, 5-min, and 10-min. These measurements were performed by using a hand-held device (NOVA Lactate Plus, Biomedical, Waltham, MA, USA) which was previously validated [25]. The device uses 0.7 µL of a whole blood sample to determine plasma lactate concentration in the range of 0.3–25.6 mmol/L within 15 s. To perform the measurement, participants’ middle or ring finger was punctured with a disposable Lancet BD puncture. Then, the puncture site was gently pressed, and the blood sample was collected. The first drop of blood was wiped off with a sterile swab, and the second drop of blood was used for lactate analysis.

### 2.5. Blood Pressure and Heart Rate Measurements

Seated systolic (SBP) and diastolic (DBP) BP were measured five times as follows: at baseline, 60-min after the ingestion of the ED/placebo drink, post-running at 1-min, 5-min, and 10-min. These measurements were performed by using an electronic sphygmomanometer (American Diagnostic Corporation, Model Advantage 6021, Hauppauge, NY, USA). When seated, participants were instructed to have their back supported, feet on the floor, and arms supported at heart level. Seated HR was also measured five times as follows; at baseline, 60-min after the ingestion of the ED/placebo drink, post-running at 1-min, 5-min, and 10-min. These measurements were performed by using a Polar H7 strap monitor (Polar H7, Polar Electro Oy, Kempele, Finland), a smartphone (iPhone SE, San Francisco, CA, USA), and a phone app (MOTIFIT, MotiFIT Fitness Inc., Moncton, NB, Canada). Participants wore a Polar strap on their chest which was synchronized to the iPhone via Bluetooth. Then, the iPhone was tightly placed on the participants’ right arm to allow HR data to be transmitted to the MOTFIT app. The collected HR data were exported to a computer software program (Microsoft Excel, Microsoft, Redmond, WA, USA) for analysis.

### 2.6. 3-Km Running Trials

Participants performed two 3-km running trials. Running was performed on a 400-m official, outdoor track. One week prior to the first trial, participants performed 3-km running familiarization trial. On experimental days, participants underwent baseline measurements and consumed the ED/placebo drink at our laboratory. Participants were instructed to arrive at the running track 30 min prior to running time. Then, they performed their usual warm-up exercises including static, dynamic stretching, and potentiating exercises. All participants were instructed to wear the same uniform and shoes during both the ED and placebo drink trials to minimize external influences. Coaches provided maximal encouragement to all participants to achieve their best performance during both trials. Weather conditions including temperature, humidity, and wind speed were recorded on both trial days. Average temperature, humidity, and wind speed were 75.18 ± 8.80 F° vs. 75.18 ± 8.66 F°, 59.1 ± 8.54% vs. 64.1 ± 13.38%, and 10.1 ± 4.25 m/s vs. 10.4 ± 3.75 m/s on the ED and placebo drink trial days, respectively.

### 2.7. ED and Placebo Drink

Participants were instructed not to drink any caffeinated drinks at least one week prior to their 1st trial and during a 7-day wash-out period. The ED (240 mL) contained 8% Calamansi juice (which is rich with vitamin C), 0.1% additive vitamin C, 10% glucose, 0.8% taurine, and 0.4% branched-chain amino acid. The placebo drink (240 mL) contained 0.02% sucralose (mimicked the sweetness of the ED), 0.05% malic acid (mimicked the taste of the ED), and 0.03% oleoresin turmeric (mimicked the color of the ED). The volume, texture, and appearance were similar across both the ED and placebo drink. To ensure the randomization and crossover design, two small papers were folded after writing the following on one side: “Red” (indicates the ED) or “Blue” (indicates the placebo drink). Then, participants picked up one of the two folded papers and were assigned to their trials accordingly. Participants, coaches, and examiners were not aware of which word matched with which drink, except one examiner who was not directly involved in the measurements. Therefore, the randomized double-blind design was well controlled. Participants consumed the ED/placebo drink 60 min prior to the 3-km running trials. Once the ED/placebo drink was provided to participants, they were instructed to drink it immediately in front of a blinded examiner who ensured that participants drank the entire drink. The time interval (i.e., 60 min) between the consumption of the drink and the start of 3-km running trials was chosen according to previous studies [26,27]. Lastly, the ED had been tested by a certified company (OATC Inc., Seoul, Korea) and was officially certified as a non-caffeinated ED by the Ministry of Food and Drug Safety in South Korea (MFDS FID-2016042480).

### 2.8. Statistical Analysis

A power analysis using G*Power program 3.1.9.2 (Heinrich-Heine-Universität, Dusseldorf, Germany) was used based on the previous study [28] to determine the sample size required to detect the difference between two dependent means (matched pairs). With an estimated power of 0.95, alpha of 0.05, a total sample of 10 in each group was required to detect an effect size of 1.3 (Actual power: 0.95). SPSS (version 25, SPSS Inc, Chicago, IL, USA) was used to perform the statistical analysis. All data were expressed as means and standard deviations. A statistical descriptive test was performed on height, weight, BMI, and body fat percentage. Kolmogorov–Smirnov test was utilized for normality evaluation of all analyzed variables. A paired *t*-test was performed to compare average performance time and speed between the two trials. A 2 × 6 (conditions × time points) repeated measures ANOVA was performed to examine interaction effects for speed by time of the 3-km running trials. A 2 × 5 (conditions × time points) repeated measure ANOVA analysis was also conducted to evaluate interaction effects for recovery variables including BLC, SBP, DBP, and HR. Bonferroni post-hoc test was applied when significant interaction or main effects were detected. Effect sizes were calculated as partial eta-squared (η^2^_p_; small ≥ 0.01, medium ≥ 0.06, large ≥ 0.14) values within measures ANOVA and Cohen’s d (small ≥ 0.02, medium ≥ 0.05, large ≥ 0.08) for paired *t*-test. Statistical significance was set a priori at *p* < 0.05.

## 3. Results

The characteristics of the participants are presented in Table 1. Seven participants reported themselves as males. Participants were 20.8 ± 1.5 years old with normal SBP (116.7 ± 8.2 mmHg), DPB (70.9 ± 12.2 mmHg), BMI (20.0 ± 2.5 kg/m^2^), and body fat percentage (9.3 ± 5.1%). All analyzed variables were normally distributed. There was no significant baseline difference in BLC, SBP, DBP, and HR between the trials. There were a few missing data from recovery outcomes including blood lactate and blood pressure (single data at recovery 1 min) due to unexpected device errors. Therefore, only completed data from all time points were included in the analysis.

**Figure 2:**
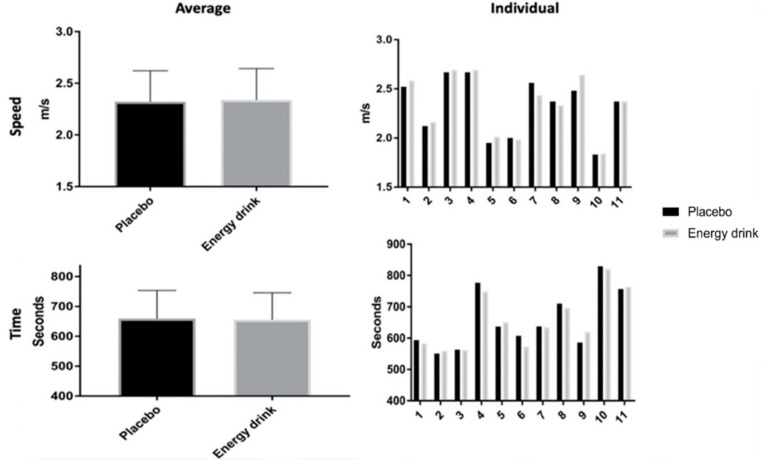
Running performance time and speed. Note. m/s: meter per second.

Figure 2 displays performance time and speed in both trials. The paired *t*-test showed no significant difference between the two trials in both average performance time and speed (t = 0.681; *p* = 0.511; Cohen’s d = 0.044 and t = 0.832; *p* = 0.425; Cohen’s d = 0.023, respectively). The average performance time for the placebo and ED trials were 658.96 ± 94.53 s and 655.04 ± 90.42 s, respectively. The average performance speed for the placebo and ED trials were 2.34 ± 0.30 s and 2.32 ± 0.30 s, respectively. The repeated ANOVA showed no interaction for trial by time effect on performance time and speed (F = 1.455; *p* = 0.221; η^2^_p_ = 0.127 and F = 0.815; *p* = 0.422, η^2^_p_ = 0.075, respectively). There also was no trial effect on both performance time and speed (F = 0.708; *p* = 0.420; η^2^_p_ = 0.066 and F = 0.692; *p* = 0.425; η^2^_p_ = 0.065, respectively). However, there was a main time effect on both performance time and speed (F = 450.69; *p* < 0.001; η^2^_p_ = 0.978 and F = 65.02; *p* < 0.001; η^2^_p_ = 0.867, respectively).

**Figure 3:**
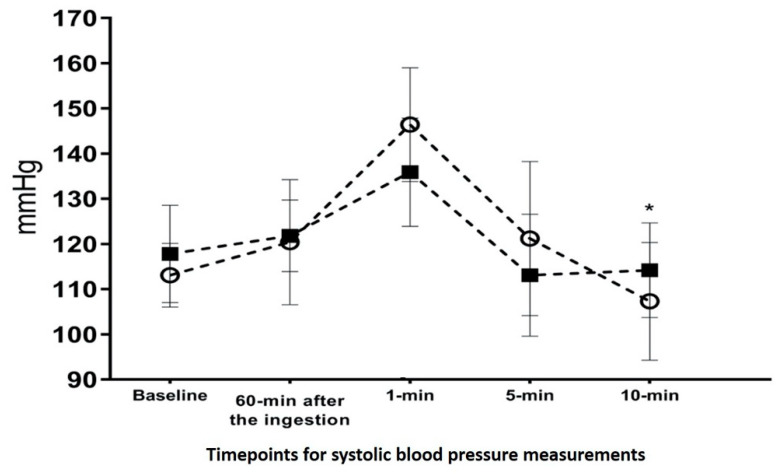
The changes of systolic blood pressure. The square indicates the placebo trial while the circle indicates the energy drink trial.* *p* < 0.05, indicates a significant change from recovery 5-min to 10 min only in the energy drink trial. mmHg: millimeters of mercury, SBP: systolic blood pressure.

Table 2 presents the changes of BLC, BP, and HR during recovery. There was no main trial or interaction effect for BLC (F = 0.117; *p* = 0.741 and F = 0.551; *p* = 0.700, respectively). However, there was a main time effect for BLC (F = 136.18; *p* < 0.001). Though there was no main trial effect (F = 1.119; *p* = 0.325) for SBP, there were main time and interaction effects (F = 28.899; *p* < 0.001 and F = 4.117; *p* = 0.010, respectively). Comparing 5-min versus 10-min post-running, SBP greatly decreased in the ED (121.2 ± 17.07 vs. 107.3 ± 13.06 mmHg) compared to the placebo trial (113.1 ± 13.48 vs. 114.2 ± 10.49 mmHg) (Figure 3). In addition, there was no main trial or interaction effect for DBP (F = 0.572; *p* = 0.469 and F = 1.544; *p* = 0.210, respectively); yet there was a main time effect for DBP (F = 3.65; *p* < 0.037). Lastly, there was no main trial or interaction effect for HR (F = 0.884; *p* = 0.372 and F = 0.722; *p* = 0.582, respectively). However, there was a main time effect for HR (F = 95.83; *p* < 0.001).

## 4. Discussion

This study examined the effect of a non-caffeinated ED that contained calamansi juice, taurine, and glucose on 3-km performance and recovery in NCAA I division distance runners. Our results revealed no significant effects of the ED on 3-km running performance (i.e., time and speed). Though the ED did not improve BLC, DBP, and HR, it significantly affected SBP reduction during recovery compared to the placebo trial.

To the best of our knowledge, this study was the first to evaluate the effect of a non-caffeinated ED that contained calamansi juice, taurine, and glucose on 3-km running performance. Though the ingredients of the ED such as glucose and taurine are believed to promote energy production which may enhance exercise performance, we did not detect improvements on any of the running performance variables. A recent meta-analysis revealed that taurine plays an essential role in EDs that induce exercise performance improvement [29]. A previous study revealed that consuming ED that contained caffeine, taurine, and glucose improved 5-km running time in healthy, recreational runners [27]. Prins et al. ED contained 80 mg caffeine with unknown concentrations of taurine and glucose. In our current study, the ED consisted of 19.2 mL taurine and 24 mL glucose. Though the minimal, beneficial concentrations of taurine and glucose in EDs remained to be examined, it is possible that these inconstant results be due to different concentrations of EDs’ ingredients. Additionally, the caffeine substance, sample characteristics, and running distance may also have roles in these equivocal findings. However, one existing study examined the acute effect of a non-caffeinated ED that contained vitamin C, taurine, and glucose on muscular (vertical jump) and anaerobic power following a simulated basketball game (SBG) in college basketball players [21]. The ED was provided to participants immediately after SBG. Then, after 20 min, the study assessments took part. The researchers demonstrated that the consumption of the ED favorably influenced vertical jump and anaerobic power comparing to a placebo drink trial. In contrast, both running time and speed (aerobic endurance) did not improve following the consumption of a non-caffeinated ED containing calamansi juice, taurine, and glucose in the current study. The ED was provided to participants 60 min prior to running. It is difficult to directly compare the efficacy of the ED on exercise performance between these two studies. Because these two studies had different study protocols such as dependent variable (anaerobic vs. aerobic performance) and subjects (basketball player vs. long-distance runner). However, it is possible that a dose–response of this ED on performance can be varied by different populations, ingestion time points, and performance variables; thus, future studies may need to be examined to find underlying mechanisms of this ED on physiological responses.

Generally, glucose is considered one of the main ingredients in EDs [30]. Glucose is added to EDs due to its properties to promote the production of a molecular unit of currency (ATP) and the ability to enhance exercise performance and recovery [31,32]. Many studies demonstrated enhanced carbohydrate availability following pre-exercise consumption of glucose which eventually enhances exercise performance [33,34,35]; however, our results showed that the pre-exercise consumption of ED that contained glucose (24 mL) did not boost running performance. A few factors may explain these inconsistent results including resting blood glucose level and concentration of consumed glucose. Generally, 30–60 g/h glucose is recommended to be consumed during endurance performance to maintain the plasma glucose level in athletes [34]. However, it is important to consider the glucose recommendation in the middle-distance running event. This is because the effectiveness of the additional glucose intake could be minor if athletes’ glycogen status is sufficient prior to the event.

This study revealed that the pre-exercise consumption of the non-caffeinated ED did not influence recovery BLC or HR. These results were consistent with the literature where many studies showed no effects of pre-exercise consumption of EDs on BLC and HR [4,27,36,37]. However, a previous study reported that similar non-caffeinated ED decreased BLC and maintained blood glucose level suggesting accelerated replenishment of fuel’s deficiency following a simulated basketball game [21]. These findings signify that the pre-exercise consumption of the ED may benefit glycemic outcomes during recovery. However, the present study did not measure other glycemic outcomes thus the hypothesis should be explored in future studies. Noteworthy, it is possible that inequivalent effects between the previous study and ours are due to differences in the nature of exercises performed (i.e., basketball that mostly relies on anaerobic capacity vs. 3-km running which mostly relies on aerobic capacity). Therefore, further research investigating the influence of these non-caffeinated EDs on aerobic vs. anaerobic performance is warranted.

Although the BLC and HR were not influenced by the ED in this study, an interesting result was observed during the recovery period. As was expected, both SBP and DBP significantly decreased following both trials through exercise-induced hypotension (PEH); however, the greater reduction was observed on SBP in the ED trial. PEH is a fall in BP that occurs following exercise (even after a single bout) and can last up to ~13 h [38,39]. Even though these reductions were expected following both trials, it was also assumed that the ED would cause greater BP reductions due to its ingredients. The ED contained vitamin C and taurine which serve as antioxidant/inflammatory agents [9,40,41]. Indeed, several studies revealed that the consumption of both vitamin C and taurine can significantly decrease BP [42,43,44]. Vitamin C functions in vascular endothelium and smooth muscle and increases vascular dilation and eventually BP reduction [45]. On the other hand, the consumption of taurine can increase endothelium-dependent and independent vasodilation [44,46]. Thus, it would be assumed the antioxidant ingredients in the ED (calamansi juice) boosted PEH. Furthermore, a previous study suggested that sustained post-exercise vasodilation, which always accompanies PEH would benefit muscle glucose uptake especially in athletes [42]. Taken together, a calamansi-containing ED may provide a benefit on SBP reduction during recovery; however, it is premature to confirm this hypothesis. Further studies are required to confirm the role of this ED on BP during recovery.

The strength of this study was systemically designed as randomized, double-blind, crossover and placebo-controlled trials. Additionally, the study was applied in a practical manner where it was performed at an official, outdoor track where running competitions are held. However, small sample size (*n* = 11) may be a limitation of the study. Additionally, the enrolled participants in our current study were NCAA Division I middle-distance runners. As such, the applicability of our findings is currently limited to this elite group of athletes, and the effects of this ED on less active or sedentary populations remain to be studied. Importantly, though the ED favorably influenced blood pressure, its effects, if any, on running performance and time were minor. Not controlling for the menstrual status also limits the generalizability of the current findings. Lastly, our study examined the acute effects only, and the chronic effects of this ED on exercise performance and recovery warrant further investigation.

## 5. Conclusions

Our study found that acute consumption of a non-caffeinated ED that contains calamansi juice, taurine, and glucose does not improve exercise performance and BLC, DBP, or HR recovery. However, this drink may be effective on SBP recovery, especially in distance runners. Nevertheless, the long-term effects of this ED remain unknown. Further studies are needed to examine the acute and chronic effects of this calamansi contained ED on exercise performance and recovery among different populations. In short, the findings of this study indicate that EDs that contain calamansi juice, taurine, and glucose may be effective to improve recovery following aerobic exercises. Yet, other non-caffeinated EDs that may enhance aerobic performance remain to be explored.

## Figures and Tables

**Table 1:** Participant Characteristics (*n* = 11).

Variables	Mean ± SD
Age (years)	20.8 ± 1.5
Height (cm)	173.8 ± 9.9
Weight (kg)	60.5 ± 10.7
Body mass index (kg/m^2^)	20.0 ± 2.5
Sum of skinfold thickness (mm)	34.6 ± 16.9
Body fat percentage (%)	9.3 ± 5.1
Systolic BP (mmHg)	116.7 ± 8.2
Diastolic BP (mmHg)	70.9 ± 12.2
Resting heart rate (beats/min)	58.7 ± 9.5
Resting lactate concentration (mmol/L)	1.67 ± 0.7

BP: blood pressure, cm: centimeter, kg/m^2^: kilogram per meter squared, mm: millimeter, mmHg: millimeters of mercury, mmol/L: millimoles per liter.

**Table 2:** Changes in blood lactate concentration, blood pressure, and heart rate during recovery.

	Trial	Baseline	Before Running	R 1 min	R 5 min	R 10 min	F-value		
							G (η^2^_p_)	T (η^2^_p_)	G × T (η^2^_p_)
BLC (mmol/L)	E	1.6 (0.63)	2 (0.63)	13.2 (2.41)	11.8 (2.72)	10.2 (2.84)	0.12 (0.014)	136.18 * (0.945)	0.55 (0.064)
	P	1.9 (0.77)	2.2 (1.21)	13 (2.18)	11.2 (1.91)	9.6 (2.12)			
Systolic (mmHg)	E	113.1 (7.10)	120.4 (13.85)	146.4 (12.61)	121.2 (17.07)	107.3 (13.06)	0.11 (0.039)	19.97 * (0.796)	5.04 ^+^ (0.326)
	P	117.8 (10.77)	121.8 (13.85)	135.9 (12.61)	113.1 (17.07)	114.2 (13.06)			
Diastolic (mmHg)	E	69.2 (8.14)	72.2 (7.28)	73.4 (13.22)	67 (7.45)	71.6 (11.81)	0.57 (0.060)	3.65 * (0.289)	1.54 (0.146)
	P	70.8 (7.54)	74.9 (5.03)	71.3 (9.18)	65.3 (8.43)	62.6 (9.90)			
Heart rate (beats/min)	E	64.3 (15.68)	86.1 (13.90)	129.2 (20.74)	119.2 (14.37)	112.4 (17.84)	0.88 (0.089)	95.38 * (0.914)	0.72 (0.074)
	P	60.4 (11.87)	88.2 (16.67)	124.3 (19.27)	112.7 (15.56)	108.7 (13.66)			

* *p* < 0.05; indicate significant time effects, ^+^ *p* < 0.05; indicate significant interaction effect between group by time; BLC: blood lactate concentration; BP: blood pressure; E: energy drink; G: group; G 237 × T: group × trial; mmHg: millimeters of mercury; mmol/L: millimoles per liter; P: placebo; T: time.
